# Maternal serum biomarkers of placental insufficiency at 24–28 weeks of pregnancy in relation to the risk of delivering small-for-gestational-age infant in Sylhet, Bangladesh: a prospective cohort study

**DOI:** 10.1186/s12884-024-06588-8

**Published:** 2024-06-10

**Authors:** Sayedur Rahman, Md. Shafiqul Islam, Anjan Kumar Roy, Tarik Hasan, Nabidul Haque Chowdhury, Salahuddin Ahmed, Rubhana Raqib, Abdullah H. Baqui, Rasheda Khanam

**Affiliations:** 1 Department of Women’s and Children’s Health, Uppsala University, Akademiska sjukhuset, Uppsala, SE- 751 85 Sweden; 2 Projahnmo Research Foundation, Banani, Dhaka, 1213 Bangladesh; 3 International Center for Diarrheal Disease Research, Bangladesh, Dhaka, Bangladesh; 4 Department of International Health, Johns Hopkins Bloomberg School of Public Health, 615 N Wolfe Street, Baltimore, MD 21205 USA

**Keywords:** Small-for-gestational-age, Biomarkers, Placental insufficiency, Pregnancy-associated plasma protein-A, Placental growth factor, Serum soluble fms-like tyrosine kinase-1, Cohort study, Bangladesh

## Abstract

**Background:**

Small-for-gestational-age (SGA), commonly caused by poor placentation, is a major contributor to global perinatal mortality and morbidity. Maternal serum levels of placental protein and angiogenic factors are changed in SGA. Using data from a population-based pregnancy cohort, we estimated the relationships between levels of second-trimester pregnancy-associated plasma protein-A (PAPP-A), placental growth factor (PlGF), and serum soluble fms-like tyrosine kinase-1 (sFlt-1) with SGA.

**Methods:**

Three thousand pregnant women were enrolled. Trained health workers prospectively collected data at home visits. Maternal blood samples were collected, serum aliquots were prepared and stored at -80℃. Included in the analysis were 1,718 women who delivered a singleton live birth baby and provided a blood sample at 24–28 weeks of gestation. We used Mann-Whitney U test to examine differences of the median biomarker concentrations between SGA (< 10th centile birthweight for gestational age) and appropriate-for-gestational-age (AGA). We created biomarker concentration quartiles and estimated the risk ratios (RRs) and 95% confidence intervals (CIs) for SGA by quartiles separately for each biomarker. A modified Poisson regression was used to determine the association of the placental biomarkers with SGA, adjusting for potential confounders.

**Results:**

The median PlGF level was lower in SGA pregnancies (934 pg/mL, IQR 613–1411 pg/mL) than in the AGA (1050 pg/mL, IQR 679–1642 pg/mL; *p* < 0.001). The median sFlt-1/PlGF ratio was higher in SGA pregnancies (2.00, IQR 1.18–3.24) compared to AGA pregnancies (1.77, IQR 1.06–2.90; *p* = 0.006). In multivariate regression analysis, women in the lowest quartile of PAPP-A showed 25% higher risk of SGA (95% CI 1.09–1.44; *p* = 0.002). For PlGF, SGA risk was higher in women in the lowest (aRR 1.40, 95% CI 1.21–1.62; *p* < 0.001) and 2nd quartiles (aRR 1.30, 95% CI 1.12–1.51; *p* = 0.001). Women in the highest and 3rd quartiles of sFlt-1 were at reduced risk of SGA delivery (aRR 0.80, 95% CI 0.70–0.92; *p* = 0.002, and aRR 0.86, 95% CI 0.75–0.98; *p* = 0.028, respectively). Women in the highest quartile of sFlt-1/PlGF ratio showed 18% higher risk of SGA delivery (95% CI 1.02–1.36; *p* = 0.025).

**Conclusions:**

This study provides evidence that PAPP-A, PlGF, and sFlt-1/PlGF ratio measurements may be useful second-trimester biomarkers for SGA.

**Supplementary Information:**

The online version contains supplementary material available at 10.1186/s12884-024-06588-8.

## Background

Small-for-gestational-age (SGA) neonates are smaller than normal for their gestational age, most often defined as birth weight less than the 10th centile adjusted by gender and gestational age at delivery [1]. SGA is a measurable proxy for fetal growth restriction (FGR), and a major contributor to global perinatal mortality and morbidity. The prevalence of SGA is higher in low- and middle-income countries (LMICs), with South Asia bearing the largest burden [2]. In LMICs, about one in five infants are born SGA (i.e., 23.3 million), and one in four neonatal deaths are among such infants [3]. SGA babies are also at increased risk of neurodevelopmental and cognitive impairments in childhood as well as adult-onset non-communicable diseases [4–6]. These risks can be averted substantially if the condition is identified early during the pregnancy [7].

Detection of SGA, however, is challenging, particularly in LMICs. The detection rates achieved through the traditional approach of diagnosing SGA in routine antenatal care settings (e.g., measurement of the symphysis-fundus height) are generally low [8, 9]. Ultrasound markers such as uterine artery (UtA) Doppler, umbilical artery pulsatility index (UA-PI), or cerebroplacental ratio (CPR) are not widely used in low-resource settings, and the predictive values are not also very good [10, 11]. Maternal plasma and serum concentrations of several angiogenesis, inflammatory, and protein biomarkers during pregnancy have been investigated for many years for identifying fetal abnormalities and adverse pregnancy outcomes. Although candidate biomarkers such as pregnancy-associated plasma protein-A (PAPP-A), placental growth factor (PlGF), and serum soluble fms-like tyrosine kinase-1 (sFlt-1) have been identified in maternal blood, their associations with and the predictive values for the risk of SGA are inconsistent across studies [12–19]. Unfortunately, no viable biomarker is available to detect SGA to date. Most of the preceding research have been screening studies in nature, conducted in hospital settings in high-income countries, and focused on identifying the optimal cut-off values of the biomarkers and assessing their performances [14, 19]. There is a scarcity of population-based studies that have longitudinally evaluated the relationships between biomarkers and the risk of SGA while taking into consideration the influence of maternal and sociodemographic factors on them, which are critical to ensure the most effective use of the biomarker measurements in SGA risk assessment.

In Bangladesh, national-level estimates on SGA are unavailable. However, a few recently published research articles have reported that the prevalence of SGA in rural Bangladeshi populations ranges from 23.4 to 50.0% [20–23]. The high burden of SGA in Bangladesh makes the country a suitable place to generate evidence to inform policy by investigating the associations between maternal serum biomarkers and SGA births. In this prospective cohort study, we aimed to investigate the association between the biomarkers of placental insufficiency during 24–28 weeks of gestation and SGA deliveries among pregnant women in a rural area of Sylhet district in Bangladesh.

## Methods

### Study design, setting, and participants

The Alliance for Maternal and Newborn Health Improvement (AMANHI) -Bangladesh established a biorepository in a cohort of pregnant women and their newborns with the aim of identifying biomarkers of adverse maternal and fetal outcomes. Between 2014 and 2018, the project enrolled 3,000 pregnant women identified through population-based surveillance in two rural sub-districts of Sylhet district in northeast Bangladesh, and followed them until 60 days postpartum. The study methodologies, cohort characteristics, and key outcomes have been described elsewhere [24, 25].

Briefly, trained community health workers (CHWs) identified pregnant women through two-monthly home visits and obtained written informed consent to participate in the study. Pregnancies were confirmed via a strip-based test at home and dated through ultrasound scans between 8 and 19 weeks of gestation in the study clinic. The CHWs collected detailed epidemiological and phenotype data from the pregnant women during antenatal and post-natal visits. Study phlebotomists collected maternal blood and urine samples twice during pregnancy (8–19 weeks, and 24–28 weeks or 32–36 weeks) and once at 42–60 days postpartum. The 2nd pregnancy sample was collected from about three-fourths of the randomly selected women. Venous blood samples were collected, centrifuged, and serum, plasma and buffy-coat aliquots were prepared and stored at -80℃ using standard procedures [25].

**Fig. 1:**
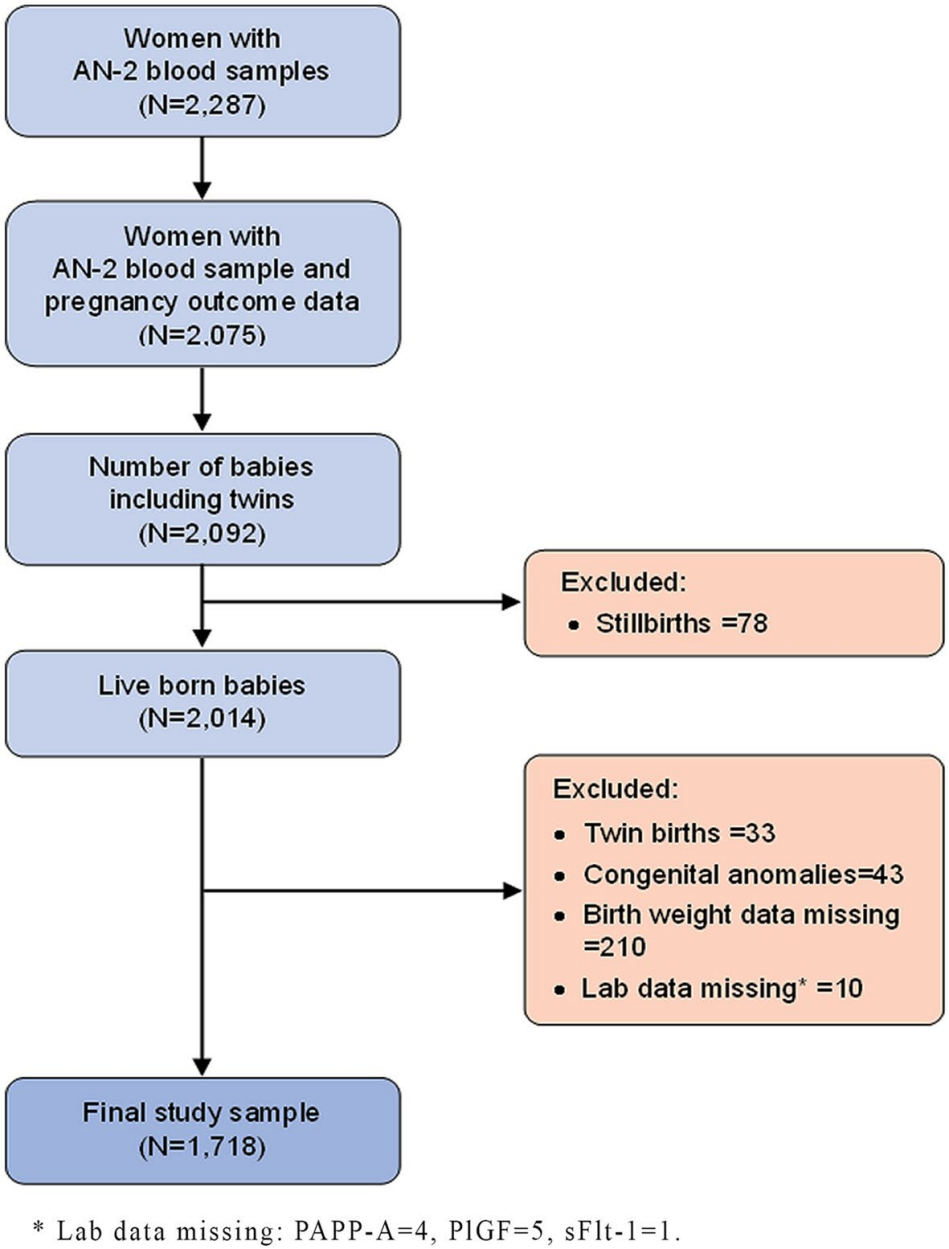
Study sample selection flow diagram

For this study, we considered all women who had a 2nd antenatal (AN-2) blood drawn at 24–28 weeks (*n* = 2,287) and had at least one antenatal and one postnatal visit and pregnancy outcome data available (*n* = 2,075). We excluded stillbirths (*n* = 78), twin pregnancies (*n* = 33), newborn congenital anomalies (*n* = 43), missing birthweight data (*n* = 210), and missing lab data (*n* = 10). Finally, a total of 1,718 samples were included in the analysis (Fig. 1).

### Biomarkers of placental insufficiency

The placenta is the channel for all maternal-fetal oxygen and nutrient exchange. Progressive deterioration in placental function, often described as “placental insufficiency”, is one of the most common causes of FGR and SGA [26]. Fetal hypoxemia and hypoglycemia induced by diminished uteroplacental and fetal-placental blood flow result in constrained fetal glycogen stores and reduced fat free mass leading to FGR [27, 28]. Placental vascular development, angiogenesis, is regulated by angiogenic proteins including PlGF as well as anti-angiogenic binding proteins such as sFlt-1 [29]. Dysregulation of these proteins is implicated in adverse maternal and fetal outcomes including pre-eclampsia (PE), SGA, and stillbirth. In SGA, circulating maternal serum level of PlGF decreases and that of sFlt-1 increases, as reported by numerous studies [16, 30–32]. Also, there is accumulating evidence of the importance of the sFlt-1/PlGF ratio as a diagnostic and prognostic marker of PE and SGA. A strong increase in sFlt-1 relative to PlGF has been found to be associated with a higher risk of SGA [15, 30, 33]. Many other studies have focused on the prediction of SGA with the help of PAPP-A, which is a placental glycoprotein produced by the syncytial trophoblast, and is believed to be involved in placental development and growth [34]. Low serum PAPP-A indicates impaired placentation and related conditions such as SGA [12, 17, 35, 36].

### Immunoassays

In our study, we collected blood samples and prepared 5–7 aliquots (500µL per aliquot) of plasma, 5–7 aliquots of serum, and one aliquot of buffy coat from the blood sample from each participating woman. The samples were stored at -80℃ using standard procedures and were analyzed at the Immunobiology, Nutrition and Toxicological Laboratory of the International Centre for Diarrhoeal Disease Research, Bangladesh (icddr, b) in 2020. Frozen serum samples from each mother were tested for biomarkers in the present study. These samples were not used in any other study hence they were not freeze-thaw samples. Serum PAPP-A, PlGF, and sFlt-1 were measured by electrochemiluminescence immunoassay (ECLIA) with Roche fully automated immunoassay analyzer Cobas e601 using commercial kits (Roche Diagnostics, GmbH, 68,305 Mannheim, Germany), and the sFlt-1/PlGF ratio was calculated for each sample. Serum levels of PAPP-A were measured in mIU/L whereas sFlt-1 and PlGF levels were measured in picograms per milliliter (pg/mL). Commercially available control human serum was run in two concentration ranges in each lot per day to monitor the accuracy and precision of the assays.

### Outcome measures

For gestational age (GA) estimation at dating ultrasound, crown-rump length (CRL) was the biometric measure of choice for fetuses less than 14 weeks (CRL < 95 mm) whereas bi-parietal diameter (BPD) and femur length (FL) were taken in addition to the CRL for fetuses at ≥ 14 to < 20 weeks of gestation [37]. GA at dating ultrasound was used to calculate GA at all subsequent study visits, at sample collection, and at birth. Infant birthweights were measured within 72 h of delivery using a digital infant scale (TANITA BD 585 Pediatric Scale; precision ± 10 gm) by study paramedics in case of hospital deliveries, and by the CHWs in case of home deliveries. SGA in this study was defined as newborns weighing < 10th centile birth weight for GA and sex with the multiethnic INTERGROWTH-21st standard [38].

### Covariates

Mother’s age was categorized into < 30 and ≥ 30 years. Mother’s education was categorized into 0–5 years and > 5 years of schooling. Parity was categorized as 0/primiparous, 1–3, and ≥ 4 children. Maternal body mass index (BMI) was categorized into underweight (< 18.5 kg/m^2^), normal (18.5 to < 25 kg/m^2^), and overweight/obese (≥ 25 kg/m^2^). Preeclampsia (PE) was determined based on expert adjudication, which took into account pregnancy induced hypertension and proteinuria. Household crowding index was created by dividing the number of persons by number of sleeping rooms and then, was categorized into ≤ 2 and > 2. Wealth index was constructed using household asset data via principal components analysis (PCA) and were divided into tertiles.

### Statistical analysis

We examined the distributions of maternal serum levels of PAPP-A, PlGF, and sFlt-1 for normality using histograms. As the univariate distributions of the biomarkers deviated from normality, we used Mann-Whitney U tests to assess whether the median concentrations of each biomarker differed between SGA and appropriate-for-gestational-age (AGA) infants. Ratio of biomarker concentrations for sFlt-1/PlGF was calculated and also compared between the SGA and AGA groups. Frequencies and distributions of baseline covariates between SGA and AGA were compared using Student’s *t*-test for comparing the means and Pearson’s chi-squared test for categorical variables.

We created the biomarker concentration quartiles, and estimated the risk ratios (RRs) and 95% confidence intervals (CIs) for SGA by quartiles separately for each biomarker. For PAPP-A and PlGF, using the highest quartile as the reference, RRs and 95% CIs for SGA among participants in each of the lower three quartiles were calculated. Conversely, for the sFlt-1 and sFlt-1/PlGF ratio, the lowest quartile group was considered as the reference group and RRs in each of the upper three quartiles were compared to the estimates in the referent category. Selection of the reference groups were guided by existing literature. Bivariate and multivariate regression were used to calculate unadjusted and adjusted RRs with 95% CIs to identify the biomarkers associated with SGA. Covariates that predicted SGA at an α level of 0.2 in the bivariate analyses were included in the multivariate regression model. We encountered the problem of non-convergence when we tried to fit the log binomial regression model hence, eventually used a modified (robust error variance) Poisson regression to estimate the adjusted risk ratios (aRR) and CIs [39, 40]. An association was considered significant if the *p*-value was < 0.05. All the analyses were performed using Stata 13.1 SE (StataCorp. 2013. Stata Statistical Software: Release 13. College Station, TX: StataCorp LP).

## Results

Selected characteristics of mother, household, and newborn and maternal biomarker concentrations are presented by AGA and SGA pregnancies in Table 1. A total of 1,718 women with singleton livebirths were studied; 927 (54%) of them delivered AGA and 791 (46%) delivered an SGA infant. The mean GA at blood sample collection was 25.5 (± 1.37) weeks, which was similar in SGA (25.6 ± 1.39) and AGA (25.5 ± 1.35) groups. The median serum concentrations of PAPP-A were nearly the same in AGA and SGA groups. The median PlGF level was significantly lower in SGA pregnancies [934 pg/mL, Interquartile Range (IQR) 613–1411 pg/mL] than that in the AGA pregnancies (1050 pg/mL, IQR 679–1642 pg/mL; *p* < 0.001). There was only a marginal difference in median sFlt-1 levels between the two groups. The median sFlt-1/PlGF ratio, however, was significantly higher among women who delivered an SGA infant (2.00, IQR 1.18–3.24) compared to those who delivered an AGA infant (1.77, IQR 1.06–2.90; *p* = 0.006).

**Table 1:** Socio-demographic, maternal & neonatal characteristics, and median biomarker values by SGA status

Characteristics	Total *N* = 1,718	AGA *n* = 927 (54%)	SGA *n* = 791 (46%)	*p*-value
**Serum biomarker concentrations**				
PAPP-A level in mIU/L (median (IQR))	75.95 (52.20 -103.9)	76.10 (53.20 -105.20)	75.40 (50.50 -102.70)	0.686
PlGF level in pg/mL (median (IQR))	995 (648–1519)	1050 (679–1642)	934 (613–1411)	< 0.001
sFlt-1 level in pg/mL (median (IQR))	1873 (1339–2595)	1896 (1362–2620)	1832 (1300–2580)	0.283
sFlt-1/PlGF ratio (median (IQR))	1.88 (1.12–3.05)	1.77 (1.06–2.90)	2.00 (1.18–3.24)	0.006
**GA at sample collection in weeks (Mean ± SD)**	25.5 (1.37)	25.5 (1.35)	25.6 (1.39)	0.746
**Mother’s age**				
< 30 years	1,500 (87.3)	809 (87.3)	691 (87.4)	0.957
≥ 30 years	218 (12.7)	118 (12.7)	100 (12.6)	
**Mother’s education**				
0–5 years	777 (45.2)	417 (45.0)	360 (45.5)	0.826
> 5 years	941 (54.8)	510 (55.0)	431 (54.5)	
**Mother’s BMI**				
Underweight (< 18.5 kg/m^2^)	575 (33.5)	264 (28.5)	311 (39.3)	< 0.001
Normal (18.5 to < 25 kg/m^2^)	1,043 (60.7)	585 (63.1)	458 (57.9)	
Overweight/Obese (≥ 25 kg/m^2^)	100 (5.8)	78 (8.4)	22 (2.8)	
**Parity**				
0/Primipara	564 (32.8)	250 (27.0)	314 (39.7)	< 0.001
1–3	980 (57.1)	570 (61.5)	410 (51.8)	
≥ 4	174 (10.1)	107 (11.5)	67 (8.5)	
**Gestational Hypertension**				
No	1,654 (96.3)	896 (96.7)	758 (95.8)	0.366
Yes	64 (3.7)	31 (3.3)	33 (4.2)	
**Gestational diabetes (GDM)**				
No	1713 (99.7)	923 (99.6)	790 (99.9)	0.242
Yes	5 (0.29)	4 (0.43)	1 (0.1)	
**Preeclampsia**				
No	1,714 (99.8)	926 (99.9)	788 (99.6)	0.245
Yes	4 (0.2)	1 (0.1)	3 (0.4)	
**Tobacco consumption**				
No (Never quit pre/during pregnancy)	1,455 (84.7)	779 (84.0)	676 (85.5)	0.413
Yes (currently sniffing/ chewing)	263 (15.3)	148 (16.0)	115 (14.5)	
**Taken iron tablets during pregnancy**				
No	502 (29.2)	280 (30.2)	222 (28.1)	0.331
Yes	1,216 (70.8)	647 (69.8)	569 (71.9)	
**Husband’s occupation**				
Govt/private/self-employed (possibly in-door)	538 (31.3)	302 (32.6)	236 (29.8)	0.222
Daily wage/farming/other (possibly outdoor)	1,180 (68.7)	625 (67.4)	555 (70.2)	
**Household crowding**^a^				
≤ 2	1,243 (72.3)	669 (72.2)	574 (72.6)	0.854
> 2	475 (27.7)	258 (27.8)	217 (27.4)	
**Household wealth status**^b^				
Poorest	582 (33.9)	298 (32.1)	284 (35.9)	0.009
Middle	583 (33.9)	301 (32.5)	282 (35.6)	
Richest	553 (32.2)	328 (35.4)	225 (28.5)	
**Place of delivery**				
Home	473 (27.5)	248 (26.7)	225 (28.5)	0.434
Facility	1,245 (72.5)	679 (73.3)	566 (71.5)	
**Mode of delivery**				
Vaginal	1,566 (91.2)	853 (92.0)	713 (90.1)	0.172
C-section	152 (8.8)	74 (8.0)	78 (9.9)	
**GA categories at birth**				
Term (≥ 37 weeks)	1,532 (89.2)	790 (85.2)	742 (93.8)	< 0.001
Preterm (< 37 weeks)	186 (10.8)	137 (14.8)	49 (6.2)	
**Sex of the baby**				
Male	842 (49.0)	456 (49.2)	386 (48.8)	0.871
Female	876 (51.0)	471 (50.8)	405 (51.2)	

Data are number (percent), unless otherwise stated

*AGA* Appropriate for gestational age, *SGA* Small for gestational age, *PAPP-A* Pregnancy-associated plasma protein A, *PlGF* Placental growth factor, *sFlt-1* Soluble fms-like tyrosine kinase, *IQR* Interquartile range, *BMI* Body mass index, *GA* Gestational age

^a^Household crowding index was created by dividing the number of persons by number of rooms in the household and then categorized into ≤ 2 and > 2

^b^Wealth index was constructed using household asset data via principal components analysis (PCA)

*P*-value calculated by Mann-Whitney U test for the biomarkers, Student’s *t*-test for comparing the means, and by Chi-squared test for the categorical variables

The majority of women (87.3%) were below 30 years of age, and more than half of the women completed primary level of schooling (Table 1). Mothers who were underweight were more likely to give birth to an SGA infant (39.3%) than an AGA infant (28.5%). Similarly, primiparous women had a higher proportion of SGA deliveries than AGA deliveries (39.7% and 27.0% respectively). Sixty-four (3.7%) mothers developed gestational hypertension; 33 (4.2%) in SGA and 31 (3.3%) in AGA pregnancies. Five (0.29%) mothers developed gestational diabetes (GDM) and 4 (0.2%) developed pre-eclampsia (PE). Nearly three-quarters of deliveries took place in the health facilities, and more than 91% were vaginal births. Babies who were born at term (≥ 37 weeks) tend to be SGA whereas babies who were preterm (< 37 weeks) were more likely to be AGA. Mother’s body mass index (BMI), parity, household wealth index, mode of delivery, and GA categories at birth (i.e., term/preterm) were associated with the infant’s AGA and SGA status in the bivariate analysis (Table 1).

Table 2 illustrates the association between the biomarker concentrations and selected characteristics of the study participants, including sociodemographic, maternal, and infant characteristics. The median serum concentration of PAPP-A was significantly higher in women under 30 years of age (76.4 mIU/L, IQR 52.9-106.1 mIU/L) than in women aged ≥ 30 years (69.6 mIU/L, IQR 45.5–94.4 mIU/L; *p* = 0.004). Similarly, higher concentrations of sFlt-1 (1892 pg/mL, IQR 1348–2650 pg/mL) and sFlt-1/PlGF ratio (1.90, IQR 1.16–3.09) were observed in women < 30 years of age than in women aged 30 years or older (sFlt-1: 1656 pg/mL, IQR 1270–2286 pg/mL, *p* = 0.002; sFlt-1/PlGF ratio: 1.72, IQR 1.00-2.79, *p* = 0.030). Compared to women who were of normal weight and overweight/obese, women who were underweight had higher serum PAPP-A, PlGF, and sFlt-1 levels (Table 2). Primiparous women had higher levels of PAPP-A, sFlt-1, and sFlt-1/PlGF ratio compared to multiparous women. Women with gestational diabetes (GDM) had significantly lower median concentration of PAPP-A (48.2 mIU/L, IQR 42.0-49.5 mIU/L) compared to the women who did not develop GDM (76.1 mIU/L, IQR 52.4-103.9 mIU/L; *p* = 0.01); however, only 5 women developed GDM. Compared to the women who delivered a baby girl, women who delivered a baby boy had significantly higher median serum levels of PAPP-A (73.0 mIU/L, IQR 49.8- 100.8 mIU/L and 78.5 mIU/L, IQR 54.1-107.8 mIU/L respectively; *p* = 0.003) and PlGF (932 pg/mL, IQR 609–1392 pg/mL and 1056 pg/mL, IQR 693–1636 pg/mL respectively; *p* < 0.001).Women who delivered a baby girl had a higher sFlt-1/PlGF ratio (2.05, IQR 1.25–3.19) compared to the women who delivered a baby boy (1.72, IQR 1.03–2.84; *p* = 0.0001).

**Table 2:** Distribution of maternal serum PAPP-A, PlGF, and sFlt-1 concentrations and sFlt-1/PlGF ratio at 24–28 weeks of pregnancy by selected characteristics of mothers and households in AMANHI-Bangladesh pregnancy cohort

Characteristics	PAPP-A			PlGF			sFlt-1			sFlt-1/PlGF ratio		
	Median	IQR	*p*-value	Median	IQR	*p*-value	Median	IQR	*p*-value	Median	IQR	*p*-value
**Mother’s age**												
< 30 years	76.4	52.9- 106.1	**0.004**	992	640–1528	0.515	1892	1348–2650	**0.002**	1.90	1.16–3.09	**0.030**
≥ 30 years	69.6	45.5–94.4		1019	685–1491		1656	1270–2286		1.72	1.00- 2.79	
**Mother’s education**												
0–5 years	75.1	53.1- 101.1	0.628	1029	653–1585	0.331	1871	1349–2630	0.832	1.88	1.08–3.16	0.828
> 5 years	76.2	51.9- 106.3		973	640–1489		1880	1323–2584		1.88	1.18–2.98	
**Mother’s BMI**												
Underweight (< 18.5 kg/m^2^)	80.7	59.6–112.0	**0.0001**	1064	670–1589	**0.020**	2011	1414–2825	**0.0001**	2.03	1.16–3.38	0.066
Normal (18.5 to < 25 kg/m^2^)	74.4	49.9- 102.1		981	641–1519		1815	1330–2511		1.84	1.12–2.99	
Overweight/Obese (≥ 25 kg/m^2^)	57.4	41.6–80.3		815	622–1242		1457	1077–1981		1.67	1.13–2.53	
**Household wealth status**												
Poorest	76.5	54.2- 105.9	0.069	1010	655–1589	0.339	1933	1354–2689	0.115	1.93	1.14–3.13	0.555
Middle	76.2	52.5- 107.3		1001	653–1551		1812	1320–2548		1.84	1.09–2.82	
Richest	74.5	49.9–99.6		981	640–1385		1852	1335–2570		1.89	1.17–3.11	
**Parity**												
0/Primipara	83.7	59.5- 119.2	**0.0001**	1028	654–1540	0.255	2083	1493–2802	**0.0001**	2.01	1.27–3.19	**0.003**
1–3	72.9	49.5–99.2		973	627–1497		1768	1267–2457		1.83	1.07–3.07	
≥ 4	68.0	47.1–94.4		1069	700–1580		1648	1309–2333		1.64	1.08–2.53	
**Gestational diabetes (GDM)**												
No	76.1	52.4- 103.9	**0.01**	1002	839–1072	0.912	1237	1089–1354	0.065	1.10	1.09–1.56	0.158
Yes	48.2	42.0- 49.5		994	646–1527		1875	1342–2595		1.89	1.13–3.05	
**Gestational hypertension**												
No	75.7	52.2- 103.9	0.893	985	598–1256	0.239	1923	1390–2711	0.871	2.08	1.43–2.84	0.225
Yes	78.4	54.2–99.8		997	648–1542		1873	1339–2593		1.85	1.11–3.05	
**Tobacco consumption**												
No (Never, Quit pre/during pregnancy)	75.8	51.5- 102.7	0.496	992	641–1490	0.160	1880	1336–2585	0.648	1.90	1.14–3.02	0.400
Yes (currently sniffing/ chewing)	76.4	53.3- 109.3		1013	670–1772		1824	1349–2689		1.76	0.97–3.30	
**Taken iron tablets during pregnancy**												
No	77.4	53.4- 106.8	0.264	992	627–1551	0.946	1902	1309–2572	0.848	1.87	1.12–3.12	0.915
Yes	75.1	51.5- 102.9		1000	653–1508		1848	1350–2613		1.88	1.13–3.02	
**GA categories at birth**												
Term (≥ 37 weeks)	75.7	52.1- 102.7	0.173	1001	653–1506	0.246	1875	1331–2613	0.635	1.87	1.12–3.03	0.217
Preterm (< 37 weeks)	78.8	54.0- 112.0		967	578–1621		1851	1458–2508		1.96	1.21–3.18	
**Sex of the baby**												
Male	78.5	54.1- 107.8	**0.003**	1056	693–1636	**< 0.001**	1829	1324–2637	0.507	1.72	1.03–2.84	**0.0001**
Female	73.0	49.8- 100.8		932	609–1392		1900	1351–2586		2.05	1.25–3.19	

Data are median serum concentrations of the biomarkers; measuring unit mIU/L for PAPP-A and pg/mL for sFlt-1 and PlGF

*PAPP-A* Pregnancy-associated plasma protein A, *PlGF* Placental growth factor, *sFlt-1* Soluble fms-like tyrosine kinase, *BMI* Body mass index, *IQR* Inter quartile range, *GA* Gestational age

*P*-value calculated by Mann-Whitney U test or Kruskal-Wallis test for the biomarker

Unadjusted and adjusted RRs for an SGA delivery according to the quartiles of biomarker values are presented in Table 3. When adjusted for potential confounding factors in the modified Poisson regression model, women with PAPP-A level in the lowest quartile showed a 25% significantly higher risk of SGA deliveries (95% CI 1.09–1.44; *p* = 0.002) compared to women in the reference group although the association was not statistically significant in the unadjusted model and the effect size was also low. A clear trend of increases in the risk of SGA delivery from the highest (reference group) to the lowest quartiles of PlGF was observed, with the increase more pronounced in the lowest quartile. The risks of SGA deliveries were significantly higher in women in the lowest (aRR 1.40, 95% CI 1.21–1.62; *p* < 0.001) and 2nd quartiles (aRR 1.30, 95% CI 1.12–1.51; *p* = 0.001) compared to those in the highest quartile of PlGF. Women with sFlt-1 levels in the highest and 3rd quartiles were at reduced risk of giving birth to an SGA infant (aRR 0.80, 95% CI 0.70–0.92; *p* = 0.002, and aRR 0.86, 95% CI 0.75–0.98; *p* = 0.028, respectively) compared to women in the reference group. In the unadjusted model, women with sFlt-1/PlGF ratios in the 3rd and highest quartiles were at 17% and 21% significantly higher risk of delivering SGA infant (RR 1.17, 95% CI 1.01–1.35; *p* = 0.041 and RR 1.21, 95% CI 1.04–1.40; *p* = 0.011 respectively). However, the associations were attenuated in strength after controlling for other covariates, and eventually was only significant in the highest quartile with 18% higher risk of SGA delivery (aRR 1.18, 95% CI 1.02–1.36; *p* = 0.025). Other independent factors that significantly predicted SGA in the multivariate regression analysis included malnourished mothers (both underweight and overweight), primiparity, poor household economic status, and preterm delivery (Supplementary tables S1- S4; see Additional file 1).

**Table 3:** Risk Ratios of SGA delivery by quartiles of biomarker concentrations (*N* = 1,718)

Variables	Unadjusted			Adjusted^†^		
	RR	95% CI	*p*-value	aRR	95% CI	*p*-value
**PAPP-A**						
Highest quartile (≥ 104.0 mIU/L)	Ref			Ref		
3rd quartile (76–103.9 mIU/L)	1.04	0.90, 1.20	0.598	1.10	0.96, 1.27	0.175
2nd quartile (52.3–75.9 mIU/L)	0.97	0.84, 1.13	0.708	1.04	0.90, 1.21	0.566
Lowest quartile (≤ 52.2 mIU/L)	1.08	0.94, 1.25	0.271	1.25	1.09, 1.44	**0.002**
**PlGF**						
Highest quartile (≥ 1520 pg/mL)	Ref			Ref		
3rd quartile (995–1519 pg/mL)	1.16	0.99, 1.36	0.067	1.16	0.99, 1.35	0.060
2nd quartile (649–994 pg/mL)	1.25	1.07, 1.45	**0.005**	1.30	1.12, 1.51	**0.001**
Lowest quartile (≤ 648 pg/mL)	1.33	1.14, 1.54	**< 0.001**	1.40	1.21, 1.62	**< 0.001**
**sFlt-1**						
Highest quartile (≥ 2596 pg/mL)	0.91	0.79, 1.05	0.217	0.80	0.70, 0.92	**0.002**
3rd quartile (1874–2595 pg/mL)	0.91	0.79, 1.05	0.184	0.86	0.75, 0.98	**0.028**
2nd quartile (1340–1873 pg/mL)	0.93	0.81, 1.07	0.321	0.92	0.81, 1.06	0.241
Lowest quartile (≤ 1339 pg/mL)	Ref			Ref		
**sFlt-1/PlGF ratio**						
Highest quartile (≥ 3.05)	1.21	1.04, 1.40	**0.011**	1.18	1.02, 1.36	**0.025**
3rd quartile (1.89–3.04)	1.17	1.01, 1.35	**0.041**	1.14	0.98, 1.31	0.084
2nd quartile (1.13–1.88)	1.02	0.88, 1.20	0.760	1.04	0.89, 1.21	0.624
Lowest quartile (≤ 1.12)	Ref			Ref		

*RR* Risk ratio, *aRR* Adjusted risk ratio, *CI* Confidence interval, *Ref* Reference category

An association is significant if *p*-value is < 0.05 (marked with bold letters)

^†^For each of the serum biomarkers, robust Poisson regression models adjusted for mother’s BMI, parity, household wealth status, mode of delivery, and GA categories at birth

## Discussion

### Main findings of the study

In this population-based cohort study of pregnant women in rural Bangladesh, we found that maternal mid-pregnancy serum concentration of PlGF and the ratio of sFlt-1/PlGF differed significantly between women who delivered an SGA infant compared to those who did not. Median serum levels of PAPP-A and sFlt-1 were similar between the two groups. We documented that very low maternal serum PAPP-A, low serum PlGF, and very high sFlt-1/PlGF ratio were associated with an SGA risk. Increased levels of sFlt-1 were associated with a reduced risk of giving birth to an SGA infant. To the best of our knowledge, this is the first study from Bangladesh that investigated the association between the biomarkers of placental insufficiency and the risk of SGA. This study provides evidence that PAPP-A, PlGF, and sFlt-1/PlGF ratio measurements may be useful second-trimester biomarkers for SGA.

### Serum levels of PAPP-A are not different between SGA and AGA mothers

There is increasing evidence that low maternal serum levels of PAPP-A in the first trimester of pregnancy are correlated with increased risk of SGA deliveries [12, 17, 18, 35, 36]. However, not many studies about mid- and late-pregnancy PAPP-A levels and SGA are available in the literature. In our study, we have measured the second trimester PAPP-A levels and found no difference in the median PAPP-A levels between the AGA and SGA groups. In multivariate regression analysis, however, women in the lowest quartile of PAPP-A levels demonstrated a significant association with SGA risk. According to literature, it remains unclear whether mid-to-late pregnancy levels of PAPP-A can predict the risk of SGA as efficiently as the early pregnancy PAPP-A does. From a case-control study, Bersinger et al. [41] reported significantly reduced PAPP-A level at 17 weeks of pregnancy but not at 25 and 33 weeks among women who delivered an SGA infant. Another study conducted by Lesmes and colleagues demonstrated that, compared to the normal outcome group, the mean PAPP-A level at 19–24 weeks was significantly reduced only among the term (delivered ≥ 37 weeks) SGAs but not among preterm (< 37 weeks) SGAs [13]. In normal pregnancies, serum levels of PAPP-A become detectable soon after implantation and increase throughout the pregnancy. The levels increase exponentially with a short doubling time during the first trimester, and then continue to rise at a slower pace until delivery [42]. In the abnormal maternal and neonatal conditions such as PE and SGA, the circulating PAPP-A in maternal blood reduce substantially but the levels may fluctuate when influenced by maternal characteristics and specific disease conditions [43]. Although the present study showed a relationship between very low PAPP-A level and SGA, the timing of blood sampling for the most effective use of this biomarker for prediction purposes merits further research.

### Decreased levels of PlGF is associated with the risk of SGA

The PlGF and sFlt-1 are the most widely studied angiogenic markers for prediction of SGA [19]. PlGF is a potent angiogenic factor that affects early placental vascular development and hence, is a key to optimizing fetal growth. We observed that the concentrations of PlGF differed significantly between the AGA and SGA groups. There was an increasing trend for the risk of SGA deliveries with decreasing quartiles of maternal serum PlGF levels. Our findings are consistent with preceding research, which reported that low maternal serum PlGF levels, not only in the second trimester [13, 31, 44] but also in the first [12, 30, 35, 45] and third trimesters of pregnancy [16], are associated with increased risk of SGA. PlGF in mid-pregnancy may have the potential to serve as a biomarker for prediction of SGA in this population.

### Higher levels of sFlt-1 reduce the risk of SGA

Pregnancies complicated by FGR/SGA exhibit decreased uterine blood flow, which limits the amount of oxygen in the placenta [46]. Reduced placental perfusion and lowered oxygen availability in SGA placentas are believed to be associated with increased production of sFlt-1 [46, 47]. Several studies have shown that circulating maternal serum levels of sFlt-1 during second and third trimesters significantly increased in SGA pregnancies [16, 31, 32]. Contrary to these reports, we have observed a decreasing trend for the risk of SGA with increasing quartiles of sFlt-1 levels in the second trimester. In two previous studies, Smith et al. [48] and Asvold et al. [30] have seen similar associations between sFlt-1 and SGA but in the first trimester of pregnancy. On the other hand, several other studies found no association between this biomarker and SGA [45, 49, 50]. All these findings indicate that the results related to sFlt-1 and SGA across the studies and settings are not consistent. Given the fact that placental angiogenesis involves a complex set of dynamic process which greatly changes as pregnancy progresses and as such it is regulated by a complex interplay between various factors, it is difficult to provide a possible explanation of the protective effects of sFlt-1 on SGA that we have observed in the present study. Our study finding may have raised questions about the hypothesis that reduced placental perfusion alone is sufficient to increase sFlt-1 level in maternal circulation, and the existing knowledge about the factors responsible for up- or downregulation of sFlt-1 in pregnancies complicated by SGA.

### sFlt-1/PlGF ratios are significantly higher in SGA mothers

We observed that women in the second, third, and highest quartiles of sFlt-1/PlGF ratio had a trend towards a higher risk of SGA delivery compared to women in the lowest quartile although the association was statistically significant only in the highest quartile with 18% higher risk of SGA. The median sFlt-1/PlGF ratio also differed significantly between the AGA and SGA groups. This finding is in accordance with several previously published studies in various settings [15, 30, 33, 45, 51]. There is emerging evidence that, in pregnancies complicated with SGA, circulating maternal serum levels of PlGF are decreased and levels of sFlt-1 are increased leading to an increased sFlt-1/PlGF ratio – which is currently being considered as a strong diagnostic and prognostic marker for prediction of adverse maternal and fetal outcomes including PE and SGA. The sFlt-1/PlGF ratio is even thought to be a better predictor of risk than either biomarker alone [51], which might have reflected in our study. The study findings provide the possibility for sFlt-1/PlGF ratio as potential biomarker for SGA in our setting.

### Strengths and limitations of the study

Strengths of this study include its population-based prospective cohort design. The main outcome variable, SGA, was determined based on gestational age dating by early pregnancy ultrasound conducted by trained sonographers and birthweights measured within 72 h after delivery by trained study workers, using globally accepted fetal growth standard. This study has some limitations. We did not have data on all possible risk factors for SGA deliveries. Although we collected data prospectively, the study might have been susceptible to recall bias. Another limitation of our study is the timing of measurement of the biomarker levels. Previous studies have reported that serum levels of the biomarkers during the first, second, and third trimester are associated with the risk of delivering SGA infant. By focusing on the biomarker levels between 24 and 28 weeks of gestation only, we might have missed critical periods in pregnancy development.

### Implications for future research

Numerous studies have demonstrated associations between the biomarkers of placental insufficiency with PE and GDM [12, 13, 18, 31, 33, 48, 49]. In our cohort, there were only 5 GDM and 4 PE cases and they were not associated with any of the biomarkers. Nonetheless, we reanalyzed the data by excluding the PE and GDM cases but that did not attenuate the observed associations. Thus, we did neither exclude them nor adjusted for them in our analysis. However, future studies in similar settings where the prevalence of PE and GDM is high should consider the influence of these factors while examining the associations between the biomarkers and SGA delivery. We did not observe any significant differences in the second trimester serum levels of PAPP-A and sFlt-1 between the groups of women who delivered an SGA infant and who did not. Also, higher levels of maternal serum sFlt-1 were found to be protective for SGA delivery, which is inconsistent with many prior studies. The reason for this conflicting result is likely multifactorial, including different study designs, different timing of measurement of the biomarkers, the prevalence of SGA in the population studied, and different geographical locations and ethnic backgrounds. Future studies should longitudinally evaluate associations of the biomarkers with the risk of SGA in the first, second and third trimesters of pregnancy to identify the best timing of blood sampling for prediction purposes. Further research should also be performed to examine how these biomarkers in combination with maternal characteristics and ultrasound and biophysical parameters can be used in predicting SGA in this population.

## Conclusions

Our results highlight that measurements of maternal serum PAPP-A, PlGF, and sFlt-1/PlGF ratio may be useful second-trimester biomarkers for predicting SGA among Bangladeshi pregnant women. Additional research is warranted to further explore the associations of these biomarkers, preferably in combination with other parameters, with the risk of SGA, as well as to identify their predictive values at different trimesters of pregnancy so that these biomarkers can be used as potential early diagnostic tools or targets for interventions to help prevent SGA newborns.

### Supplementary Information

Supplementary Material 1.

## Data Availability

The dataset used and analyzed for this manuscript will be available from the corresponding author on reasonable request.

## Abbreviations

SGA: Small-for-gestational age
FGR: Fetal growth restriction
LMICs: Low- and middle-income countries
UtA: Uterine artery
UA-PI: Umbilical artery pulsatility index
CPR: Cerebroplacental ratio
PAPP-A: Pregnancy-associated plasma protein-A
PlGF: Placental growth factor
sFlt-1: Serum soluble fms-like tyrosine kinase-1
AMANHI: Alliance for Maternal and Newborn Health Improvement
CHW: Community health workers
AN-2: 2nd antenatal visit
PE: Pre-eclampsia
icddr,b: International Centre for Diarrhoeal Disease Research, Bangladesh
ECLIA: Electrochemiluminescence immunoassay
GA: Gestational age
CRL: Crown-rump length
BPD: Bi-parietal diameter
FL: Femur length
BMI: Body mass index
PCA: Principal components analysis
AGA: Appropriate-for-gestational age
RR: Risk Ratio
CI: Confidence interval
aRR: Adjusted Risk Ratio
IQR: Interquartile Range
