# Supplementary material for: Maternal serum biomarkers of placental insufficiency at 24–28 weeks of pregnancy in relation to the risk of delivering small-for-gestational-age infant in Sylhet, Bangladesh: a prospective cohort study
Source: BMC Pregnancy Childbirth. 2024 Jun 10;24:418. doi: 10.1186/s12884-024-06588-8 (PMC11163798; doi:10.1186/s12884-024-06588-8)
Supplement: Supplementary file 1 — Supplementary Material 1. [file 12884_2024_6588_MOESM1_ESM.docx]

**Title: Maternal serum biomarkers of placental insufficiency at 24-28 weeks of pregnancy in relation to the risk of delivering small-for-gestational-age infant in Sylhet, Bangladesh: a prospective cohort study**

**Authors:** Sayedur Rahman^1^, Md. Shafiqul Islam^2^, Anjan Kumar Roy^3^, ASM Tarik Hasan^2^, Nabidul Haque Chowdhury^2^, Salahuddin Ahmed^2^, Rubhana Raqib^3^, Abdullah H. Baqui^4†^, Rasheda Khanam^4^.

^1^ Department of Women’s and Children’s Health, Uppsala University, Uppsala, Sweden

^2^ Projahnmo Research Foundation, Banani, Dhaka-1213, Bangladesh

^3^ International Center for Diarrheal Disease Research, Bangladesh, Dhaka, Bangladesh

^4^ Department of International Health, Johns Hopkins Bloomberg School for Public Health, Baltimore, Maryland, USA.

^†^Corresponding author

**Supplementary table 1.** Bivariate and multivariate regression analysis of the risk of SGA in relation to PAPP-A at 24-28 weeks of gestation

| **Variables** | **Unadjusted** | | | **Adjusted^†^** | | |
| --- | --- | --- | --- | --- | --- | --- |
|  | **RR** | **95% CI** | ***p-*value** | **aRR** | **95% CI** | ***p-*value** |
| **PAPP-A** |  |  |  |  |  |  |
| Highest quartile (≥ 104.0 mIU/L) | Ref |  |  | Ref |  |  |
| 3^rd^ quartile (76 - 103.9 mIU/L) | 1.04 | 0.90, 1.20 | 0.598 | 1.10 | 0.96, 1.27 | 0.175 |
| 2^nd^ quartile (52.3 - 75.9 mIU/L) | 0.97 | 0.84, 1.13 | 0.708 | 1.04 | 0.90, 1.21 | 0.566 |
| Lowest quartile (≤ 52.2 mIU/L) | 1.08 | 0.94, 1.25 | 0.271 | 1.25 | 1.09, 1.44 | **0.002** |
| **Mother’s age** |  |  |  |  |  |  |
| < 30 years | Ref |  |  |  |  |  |
| ≥ 30 years | 1.00 | 0.85, 1.16 | 0.957 |  |  |  |
| **Mother’s education** |  |  |  |  |  |  |
| 0–5 years | 1.01 | 0.91, 1.12 | 0.826 |  |  |  |
| > 5 years | Ref |  |  |  |  |  |
| **Mother’s BMI** |  |  |  |  |  |  |
| Underweight (<18.5 kg/m^2^) | 1.23 | 1.11, 1.36 | **<0.001** | 1.27 | 1.15, 1.40 | **<0.001** |
| Normal (18.5 to <25 kg/m^2^) | Ref |  |  | Ref |  |  |
| Overweight/Obese (≥25 kg/m^2^) | 0.50 | 0.34, 0.73 | **<0.001** | 0.54 | 0.37, 0.78 | **0.001** |
| **Parity** |  |  |  |  |  |  |
| 0/Primipara | 1.33 | 1.20, 1.48 | **<0.001** | 1.38 | 1.24, 1.53 | **<0.001** |
| 1–3 | Ref |  |  | Ref |  |  |
| ≥ 4 | 0.92 | 0.75, 1.13 | 0.420 | 0.94 | 0.77, 1.14 | 0.541 |
| **Tobacco consumption** |  |  |  |  |  |  |
| No (Never, Quit pre/during pregnancy) | Ref |  |  |  |  |  |
| Yes (currently sniffing/ chewing) | 0.94 | 0.81, 1.09 | 0.421 |  |  |  |
| **Taken iron tablets during pregnancy** |  |  |  |  |  |  |
| No | 0.95 | 0.84, 1.06 | 0.336 |  |  |  |
| Yes | Ref |  |  |  |  |  |
| **Husband’s occupation** |  |  |  |  |  |  |
| Govt/private/self-employed (possibly in-door) | Ref | Ref |  |  |  |  |
| Daily wage/farming/other (possibly outdoor) | 1.07 | 0.96; 1.20 | 0.227 |  |  |  |
| **Household crowding** |  |  |  |  |  |  |
| ≤ 2 | Ref |  |  |  |  |  |
| > 2 | 0.99 | 0.88, 1.11 | 0.854 |  |  |  |
| **Household wealth status** |  |  |  |  |  |  |
| Poorest | 1.20 | 1.05, 1.37 | **0.006** | 1.24 | 1.09, 1.41 | **0.001** |
| Middle | 1.19 | 1.04, 1.36 | **0.010** | 1.24 | 1.09, 1.41 | **0.001** |
| Richest | Ref |  |  | Ref |  |  |
| **Place of delivery** |  |  |  |  |  |  |
| Home | 1.05 | 0.93, 1.17 | 0.430 |  |  |  |
| Facility | Ref |  |  |  |  |  |
| **Mode of delivery** |  |  |  |  |  |  |
| Vaginal | Ref |  |  |  |  |  |
| C-section | 1.13 | 0.96, 1.33 | 0.153 | 1.15 | 0.98, 1.35 | 0.092 |
| **GA categories at birth** |  |  |  |  |  |  |
| Preterm (< 37 weeks) | 0.54 | 0.43, 0.70 | **<0.001** | 0.51 | 0.40, 0.65 | **<0.001** |
| Term (≥ 37 weeks) | Ref |  |  | Ref |  |  |
| **Sex of the baby** |  |  |  |  |  |  |
| Male | Ref |  |  |  |  |  |
| Female | 1.01 | 0.91, 1.12 | 0.871 |  |  |  |

RR, risk ratio; aRR, adjusted risk ratio; CI, confidence interval; Ref, reference category.

An association is significant if *p*-value is <0.05 (marked with bold letters).

^†^ Adjusted for mother’s BMI, parity, household wealth status, mode of delivery, and GA categories at birth.

**Supplementary table 2.** Bivariate and multivariate regression analysis of the risk of SGA in relation to PlGF at 24-28 weeks of gestation

| **Variables** | **Unadjusted** | | | **Adjusted^†^** | | |
| --- | --- | --- | --- | --- | --- | --- |
|  | **RR** | **95% CI** | ***p-*value** | **aRR** | **95% CI** | ***p-*value** |
| **PlGF** |  |  |  |  |  |  |
| Highest quartile (≥ 1520 pg/mL) | Ref |  |  | Ref |  |  |
| 3^rd^ quartile (995 - 1519 pg/mL) | 1.16 | 0.99, 1.36 | 0.067 | 1.16 | 0.99, 1.35 | 0.060 |
| 2^nd^ quartile (649 - 994 pg/mL) | 1.25 | 1.07, 1.45 | **0.005** | 1.30 | 1.12, 1.51 | **0.001** |
| Lowest quartile (≤ 648 pg/mL) | 1.33 | 1.14, 1.54 | **<0.001** | 1.40 | 1.21, 1.62 | **<0.001** |
| **Mother’s age** |  |  |  |  |  |  |
| < 30 years | Ref |  |  |  |  |  |
| ≥ 30 years | 1.00 | 0.85, 1.16 | 0.957 |  |  |  |
| **Mother’s education** |  |  |  |  |  |  |
| 0–5 years | 1.01 | 0.91, 1.12 | 0.826 |  |  |  |
| > 5 years | Ref |  |  |  |  |  |
| **Mother’s BMI** |  |  |  |  |  |  |
| Underweight (<18.5 kg/m^2^) | 1.23 | 1.11, 1.36 | **<0.001** | 1.26 | 1.14, 1.39 | **<0.001** |
| Normal (18.5 to <25 kg/m^2^) | Ref |  |  | Ref |  |  |
| Overweight/Obese (≥25 kg/m^2^) | 0.50 | 0.34, 0.73 | **<0.001** | 0.53 | 0.37, 0.77 | **0.001** |
| **Parity** |  |  |  |  |  |  |
| 0/Primipara | 1.33 | 1.20, 1.48 | **<0.001** | 1.35 | 1.22, 1.50 | **<0.001** |
| 1–3 | Ref |  |  | Ref |  |  |
| ≥ 4 | 0.92 | 0.75, 1.13 | 0.420 | 0.96 | 0.79, 1.16 | 0.668 |
| **Tobacco consumption** |  |  |  |  |  |  |
| No (Never, Quit pre/during pregnancy) | Ref |  |  |  |  |  |
| Yes (currently sniffing/ chewing) | 0.94 | 0.81, 1.09 | 0.421 |  |  |  |
| **Taken iron tablets during pregnancy** |  |  |  |  |  |  |
| No | 0.95 | 0.84, 1.06 | 0.336 |  |  |  |
| Yes | Ref |  |  |  |  |  |
| **Husband’s occupation** |  |  |  |  |  |  |
| Govt/private/self-employed (possibly in-door) | Ref | Ref |  |  |  |  |
| Daily wage/farming/other (possibly outdoor) | 1.07 | 0.96; 1.20 | 0.227 |  |  |  |
| **Household crowding** |  |  |  |  |  |  |
| ≤ 2 | Ref |  |  |  |  |  |
| > 2 | 0.99 | 0.88, 1.11 | 0.854 |  |  |  |
| **Household wealth status** |  |  |  |  |  |  |
| Poorest | 1.20 | 1.05, 1.37 | **0.006** | 1.24 | 1.09, 1.41 | **0.001** |
| Middle | 1.19 | 1.04, 1.36 | **0.010** | 1.24 | 1.09, 1.41 | **0.001** |
| Richest | Ref |  |  | Ref |  |  |
| **Place of delivery** |  |  |  |  |  |  |
| Home | 1.05 | 0.93, 1.17 | 0.430 |  |  |  |
| Facility | Ref |  |  |  |  |  |
| **Mode of delivery** |  |  |  |  |  |  |
| Vaginal | Ref |  |  |  |  |  |
| C-section | 1.13 | 0.96, 1.33 | 0.153 | 1.17 | 1.00, 1.37 | 0.052 |
| **GA categories at birth** |  |  |  |  |  |  |
| Preterm (< 37 weeks) | 0.54 | 0.43, 0.70 | **<0.001** | 0.51 | 0.40, 0.64 | **<0.001** |
| Term (≥ 37 weeks) | Ref |  |  | Ref |  |  |
| **Sex of the baby** |  |  |  |  |  |  |
| Male | Ref |  |  |  |  |  |
| Female | 1.01 | 0.91, 1.12 | 0.871 |  |  |  |

RR, risk ratio; aRR, adjusted risk ratio; CI, confidence interval; Ref, reference category.

An association is significant if *p*-value is <0.05 (marked with bold letters).

^†^ Adjusted for mother’s BMI, parity, household wealth status, mode of delivery, and GA categories at birth.

**Supplementary table 3.** Bivariate and multivariate regression analysis of the risk of SGA in relation to sFlt-1 at 24-28 weeks of gestation

| **Variables** | **Unadjusted** | | | **Adjusted^†^** | | |
| --- | --- | --- | --- | --- | --- | --- |
|  | **RR** | **95% CI** | ***p-*value** | **aRR** | **95% CI** | ***p-*value** |
| **sFlt-1** |  |  |  |  |  |  |
| Highest quartile (≥ 2596 pg/mL) | 0.91 | 0.79, 1.05 | 0.217 | 0.80 | 0.70, 0.92 | **0.002** |
| 3^rd^ quartile (1874 - 2595 pg/mL) | 0.91 | 0.79, 1.05 | 0.184 | 0.86 | 0.75, 0.98 | **0.028** |
| 2^nd^ quartile (1340 - 1873 pg/mL) | 0.93 | 0.81, 1.07 | 0.321 | 0.92 | 0.81, 1.06 | 0.241 |
| Lowest quartile (≤ 1339 pg/mL) | Ref |  |  | Ref |  |  |
| **Mother’s age** |  |  |  |  |  |  |
| < 30 years | Ref |  |  |  |  |  |
| ≥ 30 years | 1.00 | 0.85, 1.16 | 0.957 |  |  |  |
| **Mother’s education** |  |  |  |  |  |  |
| 0–5 years | 1.01 | 0.91, 1.12 | 0.826 |  |  |  |
| > 5 years | Ref |  |  |  |  |  |
| **Mother’s BMI** |  |  |  |  |  |  |
| Underweight (<18.5 kg/m^2^) | 1.23 | 1.11, 1.36 | **<0.001** | 1.26 | 1.14, 1.40 | **<0.001** |
| Normal (18.5 to <25 kg/m^2^) | Ref |  |  | Ref |  |  |
| Overweight/Obese (≥25 kg/m^2^) | 0.50 | 0.34, 0.73 | **<0.001** | 0.54 | 0.37, 0.78 | **0.001** |
| **Parity** |  |  |  |  |  |  |
| 0/Primipara | 1.33 | 1.20, 1.48 | **<0.001** | 1.38 | 1.24, 1.53 | **<0.001** |
| 1–3 | Ref |  |  | Ref |  |  |
| ≥ 4 | 0.92 | 0.75, 1.13 | 0.420 | 0.94 | 0.78, 1.15 | 0.561 |
| **Tobacco consumption** |  |  |  |  |  |  |
| No (Never, Quit pre/during pregnancy) | Ref |  |  |  |  |  |
| Yes (currently sniffing/ chewing) | 0.94 | 0.81, 1.09 | 0.421 |  |  |  |
| **Taken iron tablets during pregnancy** |  |  |  |  |  |  |
| No | 0.95 | 0.84, 1.06 | 0.336 |  |  |  |
| Yes | Ref |  |  |  |  |  |
| **Husband’s occupation** |  |  |  |  |  |  |
| Govt/private/self-employed (possibly in-door) | Ref | Ref |  |  |  |  |
| Daily wage/farming/other (possibly outdoor) | 1.07 | 0.96; 1.20 | 0.227 |  |  |  |
| **Household crowding** |  |  |  |  |  |  |
| ≤ 2 | Ref |  |  |  |  |  |
| > 2 | 0.99 | 0.88, 1.11 | 0.854 |  |  |  |
| **Household wealth status** |  |  |  |  |  |  |
| Poorest | 1.20 | 1.05, 1.37 | **0.006** | 1.24 | 1.09, 1.41 | **0.001** |
| Middle | 1.19 | 1.04, 1.36 | **0.010** | 1.23 | 1.08, 1.40 | **0.002** |
| Richest | Ref |  |  | Ref |  |  |
| **Place of delivery** |  |  |  |  |  |  |
| Home | 1.05 | 0.93, 1.17 | 0.430 |  |  |  |
| Facility | Ref |  |  |  |  |  |
| **Mode of delivery** |  |  |  |  |  |  |
| Vaginal | Ref |  |  |  |  |  |
| C-section | 1.13 | 0.96, 1.33 | 0.153 | 1.15 | 0.98, 1.36 | 0.081 |
| **GA categories at birth** |  |  |  |  |  |  |
| Preterm (< 37 weeks) | 0.54 | 0.43, 0.70 | **<0.001** | 0.51 | 0.40, 0.65 | **<0.001** |
| Term (≥ 37 weeks) | Ref |  |  | Ref |  |  |
| **Sex of the baby** |  |  |  |  |  |  |
| Male | Ref |  |  |  |  |  |
| Female | 1.01 | 0.91, 1.12 | 0.871 |  |  |  |

RR, risk ratio; aRR, adjusted risk ratio; CI, confidence interval; Ref, reference category.

An association is significant if *p*-value is <0.05 (marked with bold letters).

^†^ Adjusted for mother’s BMI, parity, household wealth status, mode of delivery, and GA categories at birth.

**Supplementary table 4.** Bivariate and multivariate regression analysis of the risk of SGA in relation to sFlt-1/PlGF ratio at 24-28 weeks of gestation

| **Variables** | **Unadjusted** | | | **Adjusted^†^** | | |
| --- | --- | --- | --- | --- | --- | --- |
|  | **RR** | **95% CI** | ***p-*value** | **aRR** | **95% CI** | ***p-*value** |
| **sFlt-1/PlGF ratio** |  |  |  |  |  |  |
| Highest quartile (≥ 3.05) | 1.21 | 1.04, 1.40 | **0.011** | 1.18 | 1.02, 1.36 | **0.025** |
| 3^rd^ quartile (1.89 - 3.04) | 1.17 | 1.01, 1.35 | **0.041** | 1.14 | 0.98, 1.31 | 0.084 |
| 2^nd^ quartile (1.13 - 1.88) | 1.02 | 0.88, 1.20 | 0.760 | 1.04 | 0.89, 1.21 | 0.624 |
| Lowest quartile (≤ 1.12) | Ref |  |  | Ref |  |  |
| **Mother’s age** |  |  |  |  |  |  |
| < 30 years | Ref |  |  |  |  |  |
| ≥ 30 years | 1.00 | 0.85, 1.16 | 0.957 |  |  |  |
| **Mother’s education** |  |  |  |  |  |  |
| 0–5 years | 1.01 | 0.91, 1.12 | 0.826 |  |  |  |
| > 5 years | Ref |  |  |  |  |  |
| **Mother’s BMI** |  |  |  |  |  |  |
| Underweight (<18.5 kg/m^2^) | 1.23 | 1.11, 1.36 | **<0.001** | 1.24 | 1.12, 1.37 | **<0.001** |
| Normal (18.5 to <25 kg/m^2^) | Ref |  |  | Ref |  |  |
| Overweight/Obese (≥25 kg/m^2^) | 0.50 | 0.34, 0.73 | **<0.001** | 0.55 | 0.38, 0.80 | **0.002** |
| **Parity** |  |  |  |  |  |  |
| 0/Primipara | 1.33 | 1.20, 1.48 | **<0.001** | 1.34 | 1.20, 1.48 | **<0.001** |
| 1–3 | Ref |  |  | Ref |  |  |
| ≥ 4 | 0.92 | 0.75, 1.13 | 0.420 | 0.95 | 0.78, 1.16 | 0.619 |
| **Tobacco consumption** |  |  |  |  |  |  |
| No (Never, Quit pre/during pregnancy) | Ref |  |  |  |  |  |
| Yes (currently sniffing/ chewing) | 0.94 | 0.81, 1.09 | 0.421 |  |  |  |
| **Taken iron tablets during pregnancy** |  |  |  |  |  |  |
| No | 0.95 | 0.84, 1.06 | 0.336 |  |  |  |
| Yes | Ref |  |  |  |  |  |
| **Husband’s occupation** |  |  |  |  |  |  |
| Govt/private/self-employed (possibly in-door) | Ref | Ref |  |  |  |  |
| Daily wage/farming/other (possibly outdoor) | 1.07 | 0.96; 1.20 | 0.227 |  |  |  |
| **Household crowding** |  |  |  |  |  |  |
| ≤ 2 | Ref |  |  |  |  |  |
| > 2 | 0.99 | 0.88, 1.11 | 0.854 |  |  |  |
| **Household wealth status** |  |  |  |  |  |  |
| Poorest | 1.20 | 1.05, 1.37 | **0.006** | 1.23 | 1.08, 1.40 | **0.002** |
| Middle | 1.19 | 1.04, 1.36 | **0.010** | 1.23 | 1.09, 1.40 | **0.001** |
| Richest | Ref |  |  | Ref |  |  |
| **Place of delivery** |  |  |  |  |  |  |
| Home | 1.05 | 0.93, 1.17 | 0.430 |  |  |  |
| Facility | Ref |  |  |  |  |  |
| **Mode of delivery** |  |  |  |  |  |  |
| Vaginal | Ref |  |  |  |  |  |
| C-section | 1.13 | 0.96, 1.33 | 0.153 | 1.15 | 0.98, 1.34 | 0.093 |
| **GA categories at birth** |  |  |  |  |  |  |
| Preterm (< 37 weeks) | 0.54 | 0.43, 0.70 | **<0.001** | 0.51 | 0.40, 0.64 | **<0.001** |
| Term (≥ 37 weeks) | Ref |  |  | Ref |  |  |
| **Sex of the baby** |  |  |  |  |  |  |
| Male | Ref |  |  |  |  |  |
| Female | 1.01 | 0.91, 1.12 | 0.871 |  |  |  |

RR, risk ratio; aRR, adjusted risk ratio; CI, confidence interval; Ref, reference category.

An association is significant if *p*-value is <0.05 (marked with bold letters).

^†^ Adjusted for mother’s BMI, parity, household wealth status, mode of delivery, and GA categories at birth.
